# Interleukin-21 activates natural killer cell activity against cetuximab-coated pancreatic tumor cells

**DOI:** 10.1186/2051-1426-3-S2-P233

**Published:** 2015-11-04

**Authors:** Elizabeth L McMichael, Alena Cristin Jaime-Ramirez, Kristan Guenterberg, Eric Luedke, Lakhvir Atwal, William E Carson

**Affiliations:** 1 The Ohio State University, Columbus, OH, USA

## 

Natural killer (NK) cells are large granular lymphocytes that play a critical role in anti-cancer mechanisms. These effector cells mediate antibody-dependent cellular cytotoxicity (ADCC) and produce IFN-γ in response to antibody-coated targets. The monoclonal antibody cetuximab targets the epidermal growth factor receptor (EGFR), which is over expressed in the majority of pancreatic cancers, but fails to effectively block MAP kinase activity when KRAS is concurrently mutated. Interleukin-21 (IL-21) is an immunomodulatory cytokine that stimulates the anti-tumor response of NK cells. We **hypothesized** that IL-21 treatment of NK cells would enhance their response to cetuximab-coated EGFR-positive pancreatic tumor cells, regardless of KRAS mutational status and thereby generate an effective anti-tumor response. Expression of EGFR was measured on six human pancreatic cancer cell lines. All six cell lines showed >85% expression of EGFR by flow cytometry, which was confirmed by immunoblot assay. NK cells from normal healthy donors and pancreatic cancer patients were treated overnight with IL-21 (10 ng/ml) and tested for their ability to lyse cetuximab-coated tumor cells in a standard 4-hour ^51^Cr release assay. NK cell killing of cetuximab-coated wild-type and mutant KRAS cell lines was significantly higher following NK cell IL-21 treatment, as compared to controls (p < 0.001). In response to cetuximab-coated pancreatic tumor cells, IL-21 treated NK cells secreted significantly higher levels of IFN-γ (p < 0.001), released higher levels of the chemokines IL-8, MIP1α/β, and RANTES (p < 0.001), induced greater chemotaxis of T cells (p < 0.01), and enhanced NK cell signal transduction via activation of ERK and STAT1. Treatment of mice bearing subcutaneous EGFR-positive/KRAS mutant pancreatic tumor xenographs with mIL-21 and cetuximab led to significant inhibition of tumor growth as compared to control conditions (p < 0.05). This result was confirmed in a second intraperitoneal model of pancreatic cancer. Treatment of tumor bearing mice with gemcitabine and cetuximab in combination led to only a modest reduction in tumor burden *in vivo* (as is seen in humans), but this effect was markedly enhanced by the addition of IL-21 (p < 0.05). Overall, this data suggests that cetuximab treatment in combination with IL-21 can greatly enhance NK cell effector functions and mediate a significant anti-tumor response in EGFR-positive pancreatic cancers in the presence of mutated KRAS.

